# Metagenomic and near full-length 16S rRNA sequence data in support of the phylogenetic analysis of the rumen bacterial community in steers

**DOI:** 10.1016/j.dib.2016.07.027

**Published:** 2016-07-19

**Authors:** Phillip R. Myer, MinSeok Kim, Harvey C. Freetly, Timothy P.L. Smith

**Affiliations:** a Department of Animal Science, University of Tennessee Institute of Agriculture, University of Tennessee, Knoxville, TN 37996, USA; b USDA-ARS, U.S. Meat Animal Research Center, Clay Center NE 68933^1^,^2^, USA

**Keywords:** 16S rRNA gene, MiSeq, Pacific Biosciences, Rumen microbiome

## Abstract

Amplicon sequencing utilizing next-generation platforms has significantly transformed how research is conducted, specifically microbial ecology. However, primer and sequencing platform biases can confound or change the way scientists interpret these data. The Pacific Biosciences RSII instrument may also preferentially load smaller fragments, which may also be a function of PCR product exhaustion during sequencing. To further examine theses biases, data is provided from 16S rRNA rumen community analyses. Specifically, data from the relative phylum-level abundances for the ruminal bacterial community are provided to determine between-sample variability. Direct sequencing of metagenomic DNA was conducted to circumvent primer-associated biases in 16S rRNA reads and rarefaction curves were generated to demonstrate adequate coverage of each amplicon. PCR products were also subjected to reduced amplification and pooling to reduce the likelihood of PCR product exhaustion during sequencing on the Pacific Biosciences platform. The taxonomic profiles for the relative phylum-level and genus-level abundance of rumen microbiota as a function of PCR pooling for sequencing on the Pacific Biosciences RSII platform were provided. For more information, see “*Evaluation of* 16*S rRNA amplicon sequencing using two next-generation sequencing technologies for phylogenetic analysis of the rumen bacterial community in steers”* P.R. Myer, M. Kim, H.C. Freetly, T.P.L. Smith (2016) [1].

**Specifications Table**

**Table:** 

Subject area	Biology
More specific subject area	Ruminant Microbiology
Type of data	Figures
How data was acquired	Next-generation sequencing technologies - Illumina MiSeq and Pacific Biosciences RSII instrument
Data format	Analyzed
Experimental factors	Rumen content samples were obtained from a contemporary group of steers as outlined in [1]. DNA was extracted from rumen samples as described in [1].
Experimental features	DNA was extracted from rumen samples using a repeated bead beating plus column (RBB+C) method [2]. Isolated metagenomic DNA was sheared to 350bp (Covaris, Woburn, MA) and used to create TruSeq^®^ PCR Free libraries for sequencing using the 2×150 NextSeq 500 high output kit and the Illumina NextSeq 500^®^ sequencing platform (Illumina, San Diego, CA). The pooled PCR amplicon libraries were sequenced using the Pacific Biosciences RSII instrument.
Data source location	Clay Center, NE, USA
Data accessibility	Data is within this article and raw ruminal MiSeq sequence data is available from the NCBI Sequence Read Archive (SRA Accession SRP047292). Additional descriptive information is associated with NCBI BioProject PRJNA261425. http://www.ncbi.nlm.nih.gov/bioproject/PRJNA261425/

**Value of the data**

• Additional consideration of primer and platform-specific biases associated with amplicon next-generation sequencing that may confound data and its interpretation.
• Further evaluation of sequencing depth of ruminal metagenomic DNA from steers.
• Greater understanding of potential PCR product exhaustion during sequencing on resultant taxonomic analyses.

## 1 Data

**Fig. 1:**
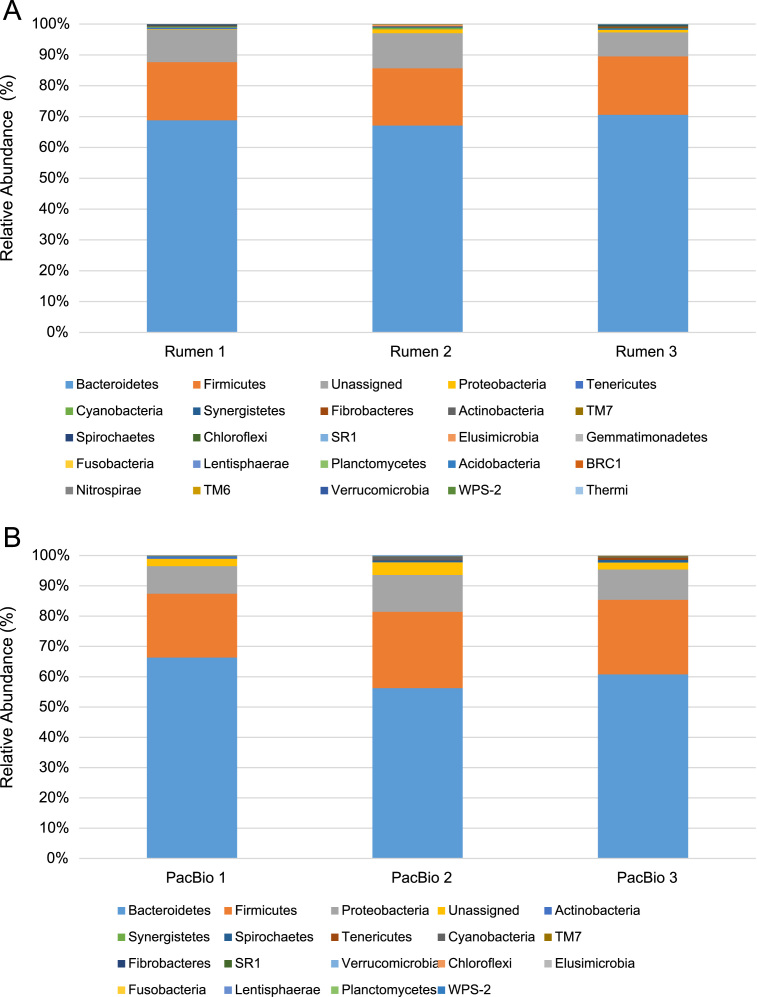
The taxonomic profiles for the relative phylum-level abundances of each sample, generated by Miseq (A) and PacBio (B) sequencing platforms, classified by representation at >0.1% of total sequences. Taxonomic composition of the ruminal microbiota among the samples was compared based on the relative abundance (reads of a taxon/total reads in a sample).

**Fig. 2:**
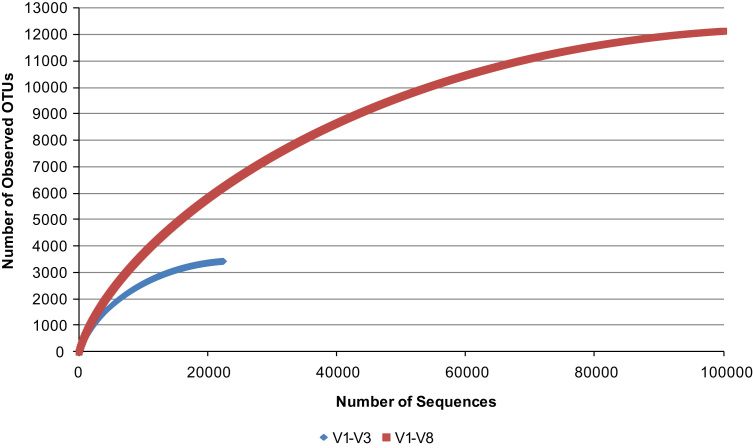
Rarefaction curves of operational taxonomic units (OTUs; ≥97% sequence similarity) for V1–V3 and V1–V8 mapped reads from metagenomic DNA.

**Fig. 3:**
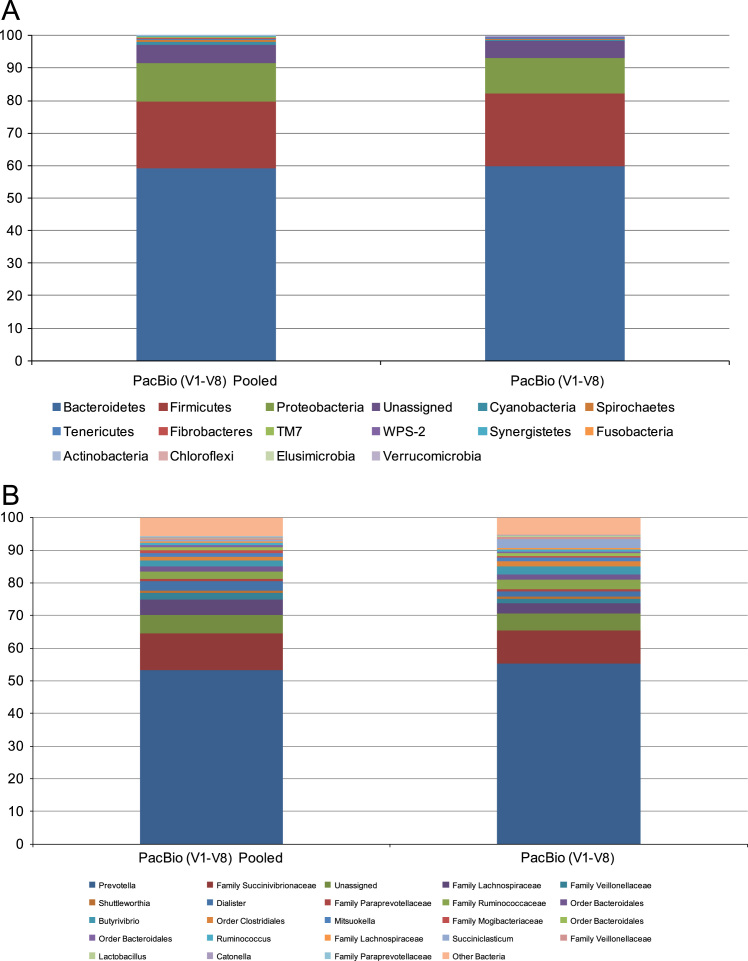
The taxonomic profiles for the relative phylum-level (A) and genus-level (B) abundance of rumen microbiota classified by representation at ≥1% of total sequences as a function of PCR pooling. Taxonomic composition of the ruminal microbiota between the two treatments was compared based on the relative abundance (reads of a taxon/total reads in a sample).

Three figures are presented. Fig. 1 contains individual animal, relative ruminal microbial abundance data from rumen samples selected from feed efficient steers (ADG_Greater_−ADFI_Less_), and the 3 with least variability among the 8 samples (animals) in the group [1], [3]; Fig. 2 depicts the data from the calculated rarefaction curves from metagenomic DNA mapped to consensus 16S rRNA V1–V3 and V1–V8 regions; Fig. 3 contains relative ruminal microbial abundance data from rumen samples regarding the reduced PCR amplification of the 16S rRNA V1–V8 hypervariable regions and pooling of the amplification products in order to determine any effects on taxonomic classification and analysis. A complete description of the data and methods is presented elsewhere [1].

## 2 Experimental design, materials and methods

### 2.1 Experimental design and rumen sampling

This experiment was approved by the U.S. Meat Animal Research Center Animal Care and Use Committee. Feed efficiency was determined as referenced by Myer et al. [3], and utilized. Three steers displaying an equivalent feed efficiency phenotype (ADG_Greater_−ADFI_Less_) and with the least deviation among each other were selected and sampled for the study.

### 2.2 DNA extraction, amplification and sequencing

DNA was extracted from rumen samples using a repeated bead beating plus column (RBB+C) method [2]. Rumen content samples were analyzed similar to Myer et al. [3]. Additionally, isolated metagenomic DNA was sheared to 350 bp (Covaris, Woburn, MA) and used to create TruSeq^®^ PCR Free libraries for sequencing using the 2×150 NextSeq 500 high output kit and the Illumina NextSeq 500^®^ sequencing platform (Illumina, San Diego, CA). Polymerase chain reaction (PCR) amplification and DNA library preparation of the V1–V8 region was performed using universal primers 27F (5′-AGAGTTTGATCCTGGCTCAG) and 1392R (5′-GACGGGCGGTGTGTAC) for the Pacific Biosciences instrument. Reduced ampliﬁcation consisted of 15 cycles, with an annealing temperature of 58 °C. PCR products were pooled for sequencing on the Pacific Biosciences RSII platform.

### 2.3 Sequence read processing and analysis

All sequences were processed using the QIIME-1.9.1 software package [4] and Mothur version 1.36.1 [5], as well as the Ribosomal RNA database project′s pyrosequencing pipeline [6] for rarefaction analysis. Pacific Biosciences reads were parsed so that quality scores of zero were interpreted as corresponding to an ambiguous base call, and then filtered for quality (≥Q30) using Mothur. Sequences that contained read lengths shorter than 1200 bp were removed. Read directionality was checked and corrected where necessary. For all reads, homopolymers >7 were discarded and chimeric sequences were checked using ChimeraSlayer [7].

Shotgun metagenomic reads were cleaned and mapped against consensus 16S rRNA V1–V3 and V1–V8 sequences, yielding 23,379 and 103,064 reads, respectively. Reads mapping to the respective variable regions were used for analysis and classification using the Greengenes 16S rRNA Gene Database, 13_8 release.
