# Supplementary material for: Metagenomic and near full-length 16S rRNA sequence data in support of the phylogenetic analysis of the rumen bacterial community in steers
Source: Data Brief. 2016 Jul 19;8:1048–53. doi: 10.1016/j.dib.2016.07.027 (PMC4969246; doi:10.1016/j.dib.2016.07.027)
Supplement: Supplementary file 1 — Supplementary material [file mmc1.docx]

**Declaration of interest** 

Conflicts of interest: none
